# 0.3-V Voltage-Mode Versatile First-Order Analog Filter Using Multiple-Input DDTAs

**DOI:** 10.3390/s23135945

**Published:** 2023-06-26

**Authors:** Montree Kumngern, Fabian Khateb, Tomasz Kulej, Pavel Steffan

**Affiliations:** 1 Department of Telecommunications Engineering, School of Engineering, King Mongkut’s Institute of Technology Ladkrabang, Bangkok 10520, Thailand; montree.ku@kmitl.ac.th; 2 Department of Microelectronics, Brno University of Technology, Technická 10, 616 00 Brno, Czech Republic; steffan@vutbr.cz; 3 Faculty of Biomedical Engineering, Czech Technical University in Prague, nám. Sítná 3105, 272 01 Kladno, Czech Republic; 4 Department of Electrical Engineering, Brno University of Defence, Kounicova 65, 662 10 Brno, Czech Republic; 5 Department of Electrical Engineering, Czestochowa University of Technology, 42-201 Czestochowa, Poland; kulej@el.pcz.czest.pl

**Keywords:** differential difference transconductance amplifier, analog filter, voltage-mode circuit, low-voltage low-power circuit

## Abstract

This paper presents a versatile first-order analog filter using differential difference transconductance amplifiers (DDTAs). The DDTA employs the bulk-driven (BD) multiple-input MOS transistors technique (MI-MOST) operating in the subthreshold region. This results in low-voltage and low-power operational capability. Therefore, the DDTA, designed using 130 nm CMOS technology from UMC in the Cadence environment, operates with 0.3 V and consumes 357.4 nW. Unlike previous works, the proposed versatile first-order analog filter provides first-order transfer functions of low-pass, high-pass, and all-pass filters within a single topology. The non-inverting, inverting, and voltage gain of the transfer functions are available for all filters. Furthermore, the proposed structure provides high-input and low-output impedance, which is required for voltage-mode circuits. The pole frequency and voltage gain of the filters can be electronically controlled. The total harmonic distortion of the low-pass filter was calculated as −39.97 dB with an applied sine wave input signal of 50 mV_pp_@ 50 Hz. The proposed filter has been used to realize a quadrature oscillator to confirm the advantages of the new structure.

## 1. Introduction

The second-generation current conveyor (CCII) was introduced in [1] as a single active building block, namely containing a voltage follower between the y- and x-terminals and a current follower between the x- and z-terminals. To increase the performance of the CCII for handling differential input signals, a differential difference current conveyor (DDCC) was proposed [2]. A DDCC includes the advantages of both CCII and the differential difference amplifier (DDA) [3] within a single circuit. One such advantage is a high input impedance and arithmetic operation capability, which is valuable for voltage-mode circuits. However, the circuits based on DDCCs lack electronic tuning capability. Thus, the next phase of development of DDCCs has been to obtain electronic tuning capability, for example, as a differential difference current conveyor transconductance amplifier (DDCCTA) [4], differential difference transconductance amplifier (DDTA) [5], or differential voltage current conveyor transconductance amplifier (DVCCTA) [6]. Recently, the DDTA has been designed to operate with a low power supply and low power consumption and has been successfully utilized for various applications with analog filters [7,8,9,10,11,12].

First-order filters are single-pole circuits that are widely used in signal processing. There are three filtering functions to realize first-order transfer functions: low-pass (LP), high-pass (HP), and all-pass (AP) filters. The first-order LP and HP filters can be used as subcircuits to realize high-order filters [13,14,15,16], while the first-order AP filters are an important block of high-quality (Q) band-pass (BP) filters [17], sinusoidal oscillators [18], time delays [19], group-delay or phase equalizers [20].

Universal first-order filters are topologies that can realize the first-order transfer functions of LP, HP, AP filters into a single circuit. There are many universal first-order filters available in open literature [21,22,23,24,25,26,27,28,29,30,31,32,33,34,35,36,37,38,39,40,41,42,43,44,45,46,47,48,49,50,51,52,53,54,55]. Nowadays, the topic of the first-order universal filter is an interesting area for research. Considering the input and output signals of universal first-order filters in [21,22,23,24,25,26,27,28,29,30,31,32,33,34,35,36,37,38,39,40,41,42,43,44,45,46,47,48,49,50,51,52,53,54,55], these topologies can be classified as current-mode (CM) filters [21,22,23,24,25,26,27,28,29,30,31,32,33,34,35], voltage-mode (VM) filters [36,37,38,39,40,41,42,43,44,45,46,47,48,49,50,51], mixed-mode (MM) filters [52,53,54,55]. Focusing on the CM filters, the circuits in [21,29] offer maximum first-order transfer functions of LP, HP, AP filters. With a suitable choice of the output currents, the filters in [21,29] offer both non-inverting and inverting transfer functions of LP, HP, AP filters, thus obtaining six transfer functions. However, the gain of the transfer functions cannot be controlled. Considering the VM filters in [36,37,38,39,40,41,42,43,44,45,46,47,48,49,50,51], the filters in [41,43,44,45,46] offer the voltage gain of the transfer functions, but these filters can control the gain only for a few transfer functions, such as LP and HP filters.

The MM filters are topologies that offer four types of transfer functions in a single circuit: VM, CM, trans-admittance mode (TAM), and trans-impedance mode (TIM). Considering MM filters in [52,53,54,55], the filters in [52,53,54] offer four types of transfer functions (CM, VM, TIM, TAM), but each type offers only three transfer functions of LP, HP, and AP filters.

In this work, a voltage-mode versatile first-order analog filter based on DDTA as an active element is presented. The work shows that the multiple-input DDTA (MI-DDTA) can offer an easy choice for voltage-mode first-order filters. The MI-DDTAs with 0.3 V and 357.4 nW consumption were used, with the bulk-driven (BD) technique of multiple-input MOS transistors (MI-MOST) operating in the subthreshold region. Unlike previous works, the proposed versatile first-order analog filter can realize both non-inverting and inverting transfer functions of LP, HP, and AP filters in a single circuit. In addition, the voltage gain of all transfer functions can be controlled. The proposed structure provides high-input and low-output impedance, which is ideal for voltage-mode circuits. Furthermore, both the pole frequency and voltage gain of all filters can be controlled electronically. The proposed filter has been used to realize a quadrature oscillator to confirm the advantages of the new structure.

## 2. Proposed Circuit

### 2.1. CMOS Structure of the Multiple-Input DDTA

**Figure 1:**
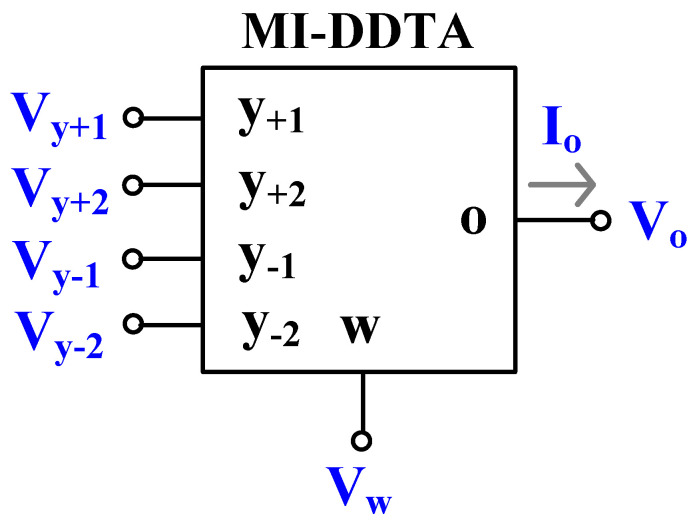
Electrical symbol of the MI-DDTA.

Figure 1 shows the electrical symbol of the DDTA. The y- and o-terminals possess a high impedance level, while the w-terminal possesses a low impedance level. The addition and subtraction voltages at the w-terminal (*V_w_*) are transformed to a current at the o-terminal by the transconductance $g_{m}$. The port characteristic of the DDTA can be expressed as:

(1) $$\left.\begin{array}{c} V_{w}=V_{+1}+V_{+2}-V_{-1}-V_{-2}\\ I_{o}=g_{m}V_{w} \end{array}\right\}$$

**Figure 2:**
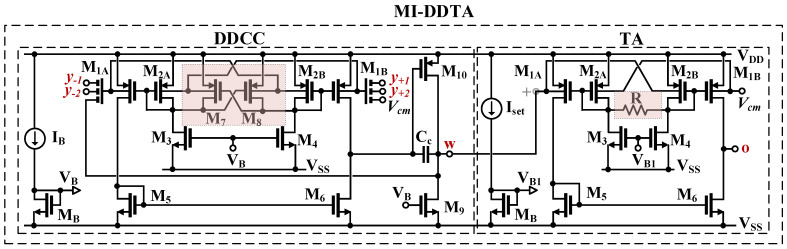
CMOS implementation of the MI-DDTA.

**Figure 3:**
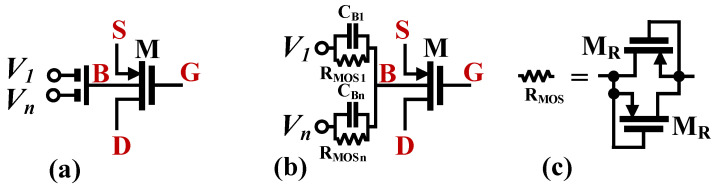
MI-BD MOST: (**a**) symbol, (**b**) realization, (**c**) possible implementation of R_MOS_.

The CMOS structure of the LV LP DDTA was first presented in [10]. The circuit, as shown in Figure 2, consists of two main blocks: a differential-difference current conveyor (DDCC) and a transconductance amplifier (TA). The DDCC can be considered as a two-stage amplifier with a differential difference input stage (M_1_–M_8_) and a second stage based on the transistor (M_10_) operating in a common source configuration. The capacitance *C*_*C*_ is used for frequency compensation. The input stage of the DDCC is based on a bulk-driven non-tailed differential pair with partial positive feedback [56,57]. The non-tailed architecture allows operation at very low supply voltages, while providing good common-mode and power supply rejection ratios (CMRR and PSRR, respectively) [56,57]. The BD technique achieves a large common-mode range, which is usually rail-to-rail. In order to improve the voltage gain of the differential amplifier, partial positive feedback (PPF) is introduced by the cross-coupled pair of transistors, M_7_ and M_8_. The transistors generate negative conductances, which partially compensate for the total conductances at the gate/drain nodes of M_2A_ and M_2B_, thus increasing the voltage gain of the whole input stage. The differential difference capability is realized using an MI-BD technique, i.e., using an additional capacitive voltage summing circuit. A particular realization of the MI-BD transistors is shown in Figure 3. The capacitors (*C*_*Bi*_) form a voltage divider/voltage summing circuit. In a passband, their impedances are much lower than the resistances of the large resistors (R_MOS_). The resistors are realized as an anti-parallel connection of two minimum-size MOS transistors operating in a cut-off region (Figure 3c), which are used to provide proper biasing of the bulk terminals of the input MOS transistors for DC. This solution provides differential difference capability without using a second transistor pair, thus saving dissipation power and simplifying the overall structure. The voltage *V_W_* can then be expressed as:

(2) $$V_{w}=\frac{A_{v}}{1+A_{v}}\left(V_{+1}+V_{+2}-V_{-1}-V_{-2}\right)$$

where the open-loop gain *A_v_* can be expressed as:

(3) $$A_{v}=\beta \cdot \frac{2g_{mb1}}{1-m}(r_{ds1}||r_{ds6})g_{m10}\left(r_{ds9}||r_{ds10}\right)$$

where β is the voltage gain of the input capacitive divider. Assuming equal capacitances, *C*_*Bi*_ is equal to 1/3, and $m=g_{m7,8}/g_{m2A,B}$ is the coefficient associated with PPF. Note that for stable operation, the value of m should always be less than unity. In the proposed design, m=0.75.

The output resistance of the DDCC, seen from the w terminal, can be expressed as:

(4) $$r_{outW}=\frac{1}{\beta \cdot \frac{2g_{mb1}}{1-m}(r_{ds1}||r_{ds6})g_{m10}}$$

and is typically 20–30 times lower than $1/g_{m10}$.

The second block of the proposed DDTA, namely the transconductance amplifier, was originally proposed and verified experimentally in [58]. The circuit can be seen as a non-tailed differential pair (M_1_–M_6_), with an additional linearization resistor *R*. Assuming M_1_ = M_2_, the circuit transconductance can be expressed as [58]:

(5) $$g_{m}=2g_{mb1,2}\frac{R+\frac{1}{g_{m1,2}}}{R+\frac{2}{g_{m1,2}}}$$

The circuit shows optimum linearity when the following condition is satisfied [58]:

(6) $$R=\frac{1}{g_{m1,2}}$$

However, the circuit linearity remains good even for relatively large differences between *R* and $1/g_{m1,2}$. This enables the tuning of the transconductance of the TA with the biasing current I_set_. The voltage gain of the TA can be expressed as:

(7) $$A_{VTA}=g_{m}\left(r_{ds1}||r_{ds6}\right)$$

Due to the non-cascoded structure, its value is relatively low but sufficient for the proposed application.

### 2.2. Proposed Voltage-Mode First-Order Versatile Analog Filter

**Figure 4:**
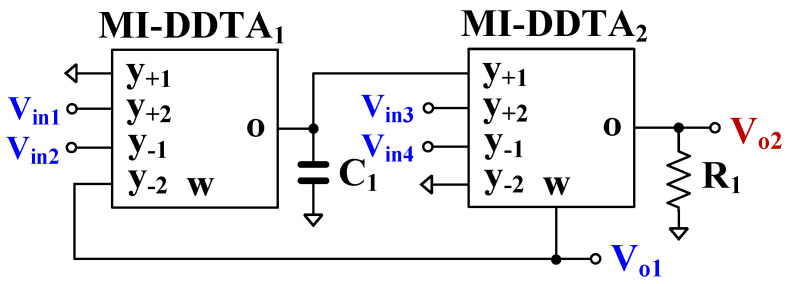
Proposed voltage-mode first-order universal analog filter.

Figure 4 shows the proposed voltage-mode first-order analog filter employing two MI-DDTAs, one grounded capacitor *C*_1_ and one grounded resistor, *R*_1_. The *V*_*in*1_, *V*_*in*2_, *V*_*in*3_, and *V*_*in*4_ are the input voltages, while *V*_*o*1_ and *V*_*o*2_ are the output voltages. The inputs, *V*_*in*1_, *V*_*in*2_, *V*_*in*3_, and *V*_*in*4_, are applied via the high-impedance level (y-terminal) of the MI-DDTA, while the output *V*_*o*1_ is available at the low-impedance level (w-terminal) of the MI-DDTA. Thus, the proposed filter possesses high-input and low-output impedances.

From Figure 4, using (1) and nodal analysis, the outputs *V*_*o*__1_ and *V*_*o*__2_ can be expressed, respectively, as:

(8) $$V_{o1}=\frac{sC_{1}\left(V_{in3}-V_{in4}\right)+g_{m1}\left(V_{in1}-V_{in2}\right)}{sC_{1}+g_{m1}}$$

(9) $$V_{o2}=g_{m2}R_{1}\left(\frac{sC_{1}\left(V_{in3}-V_{in4}\right)+g_{m1}\left(V_{in1}-V_{in2}\right)}{sC_{1}+g_{m1}}\right)$$

The LP, HP, AP filtering functions can be obtained by suitably applying the input voltages to the circuit. The variant filtering functions of first-order filters are shown in Table 1. It is obvious that the non-inverting and inverting transfer functions of LP, HP, AP filters can be obtained in a single circuit without the need for an inverted input signal. The voltage gain of all transfer functions is obtained at the output *V_o_*_2_, which can be controlled by $g_{m2}R_{1}$. The electronic tuning capability is obtained by varying $g_{m2}$. However, since this output node does not have a low-impedance level, a buffer circuit maybe required if a low-impedance load is connected.

The pole frequency of all transfer functions can be expressed by:

(10) $$\omega_{o}=\frac{g_{m1}}{C_{1}}$$

The pole frequency can be controlled by $g_{m1}$. Therefore, the voltage gain and the pole frequency can be electronically controlled.

### 2.3. Non-Ideality Analysis

The non-ideal characteristics of MI-DDTA can be considered as [11]:

(11) $$\left.\begin{array}{c} V_{w}=\beta_{+1j}V_{+1}+\beta_{+2j}V_{+2}-\beta_{-1j}V_{-1}-\beta_{-2j}V_{-2}\\ I_{o}=g_{mnj}V_{w} \end{array}\right\}$$

where, for the *j*-th DDTA, $\beta_{+1j}$ is the voltage gain between $V_{+1}$ and $V_{w}$, $\beta_{+2j}$ is the voltage gain between $V_{+2}$ and $V_{w}$, $\beta_{-1j}$ is the voltage gain between $V_{-1}$, and $V_{w}$, $\beta_{-2j}$ is the voltage gain between $V_{-2}$ and $V_{w}$, of *j*-th DDTA, and $g_{mnj}$ is the non-ideal transconductance of the *j*-th MI-DDTA. Ideally, the voltage gains, $\beta_{+1}$, $\beta_{-2}$, $\beta_{-1}$, $\beta_{-2}$, are equal to unity. However, these voltage gains may sightly deviate from ideality and affect the transfer functions of the proposed filter.

For the DDTA operating near the cut-off frequency, $g_{mnj}$ can be approximated by [11]:

(12) $$g_{mnj}=g_{mj}\left(1-\mu_{j}s\right)$$

where $\mu_{j}=1/\omega_{gj}$, and $\omega_{gj}$ denotes the first pole of the *j*-th MI-DDTA.

Using (11), (8) and (9) can be rewritten, respectively as:

(13) $$V_{o1}=\frac{sC_{1}\left(\beta_{+22}V_{in3}-\beta_{-12}V_{in4}\right)+g_{mn1}\left(\beta_{+21}\beta_{+12}V_{in1}-\beta_{-11}\beta_{+12}V_{in2}\right)}{sC_{1}+\beta_{-21}\beta_{+12}g_{mn1}}$$

(14) $$\begin{array}{l} V_{o2}\\ =g_{mn2}R_{1}\left(\frac{sC_{1}\left(\beta_{+22}V_{in3}-\beta_{-12}V_{in4}\right)+g_{mn1}\left(\beta_{+21}\beta_{+12}V_{in1}-\beta_{-11}\beta_{+12}V_{in2}\right)}{sC_{1}+\beta_{-21}\beta_{+12}g_{mn1}}\right) \end{array}$$

The non-ideal transfer functions of LP, HP, AP filters can be expressed by:

(15) $$\frac{V_{o1}}{V_{in1}}=\frac{\beta_{+21}\beta_{+12}g_{mn1}}{sC_{1}+\beta_{-21}\beta_{+12}g_{mn1}}$$

(16) $$\frac{V_{o1}}{V_{in2}}=-\frac{\beta_{-11}\beta_{+12}g_{mn1}}{sC_{1}+\beta_{-21}\beta_{+12}g_{mn1}}$$

(17) $$\frac{V_{o1}}{V_{in3}}=\frac{sC_{1}\beta_{+22}}{sC_{1}+\beta_{-21}\beta_{+12}g_{mn1}}$$

(18) $$\frac{V_{o1}}{V_{in4}}=-\frac{sC_{1}\beta_{-12}}{sC_{1}+\beta_{-21}\beta_{+12}g_{mn1}}$$

Letting $V_{in2}=V_{in3}=V_{in}$, we obtain:

(19) $$\frac{V_{o1}}{V_{in}}=\frac{sC_{1}\beta_{+22}-\beta_{-11}\beta_{+12}g_{mn1}}{sC_{1}+\beta_{-21}\beta_{+12}g_{mn1}}$$

Letting $V_{in1}=V_{in4}=V_{in}$, we obtain:

(20) $$\frac{V_{o1}}{V_{in}}=-\frac{sC_{1}\beta_{-12}-\beta_{+21}\beta_{+12}g_{mn1}}{sC_{1}+\beta_{-21}\beta_{+12}g_{mn1}}$$

Using (12), the denominator of all transfer functions (*D*(*s*)) can be expressed by:

(21) $$D\left(s\right)=sC_{1}\left(1-\frac{\beta_{-21}\beta_{+12}\mu_{1}g_{m1}}{C_{1}}\right)+\beta_{-21}\beta_{+12}g_{m1}$$

The non-ideal transconductance can be made negligible by satisfying:

(22) $$\frac{\beta_{-21}\beta_{+12}\mu_{1}g_{m1}}{C_{1}}\ll 1$$

If the non-ideal behavior of the DDTA operating at a high frequency is also considered, the parallel connection of parasitic resistance (*R*_*o*_) and parasitic capacitance (*C*_*o*_) at the o-terminal should be taken in account (*R*_*o*_//*C*_*o*_) [43]. In consideration of the non-ideal behavior of the DDTA given by *R*_*o*_//*C*_*o*_, the pole frequency of Figure 4 obtained from (21), while satisfying (22), can be determined by:

(23) $$\omega_{on}=\frac{\beta_{-21}\beta_{+12}g_{m1}}{C_{1}^{’}}$$

where $C_{1}^{’}=C_{1}+C_{o1}$ and $R_{o1}\gg 1/g_{m1}$, $C_{o1}$ and $R_{o1}$ are the parasitic capacitance and resistance at the o-terminal of MI-DDTA_1_.

## 3. Application Example

**Figure 5:**
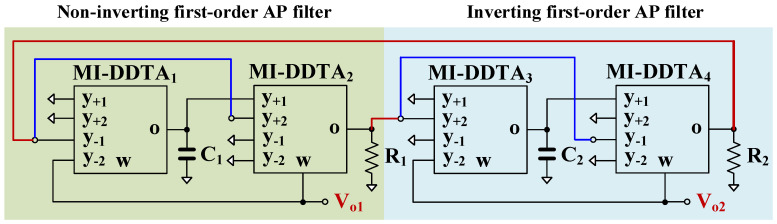
Proposed sinusoidal oscillator using first-order AP filters.

To demonstrate the advantages of the proposed versatile filter, two first-order AP filters, namely non-inverting and inverting AP filters based on the same topology, have been used to realize a sinusoidal oscillator as shown in Figure 5.

Using Table 1 and the row for AP filters, the loop gain (*LG*) of the oscillator can be given as:

(24) $$LG\left(s\right)=-g_{m2}R_{1}g_{m4}R_{2}\left(\frac{sC_{1}-g_{m1}}{sC_{1}+g_{m1}}\right)\left(\frac{sC_{1}-g_{m3}}{sC_{1}+g_{m3}}\right)$$

Letting $g_{m1}=g_{m3}=g_{mf}$, $C_{1}=C_{2}=C_{f}$, and *LG* = 1, the characteristics of the oscillator can be expressed by:

(25) $$g_{m2}R_{1}g_{m4}R_{2}{\left(\frac{sC_{f}-g_{mf}}{sC_{f}+g_{mf}}\right)}^{2}=0$$

The condition of oscillation (CO) can be given by:

(26) $$g_{m2}R_{1}g_{m4}R_{2}=1$$

The frequency of oscillation (FO) can be given as:

(27) $$\omega_{o}=\left(\frac{g_{mf}}{C_{f}}\right)\tan\left(\frac{\pi }{4}\right)$$

From (15) and (26), the CO can be controlled electronically by $g_{m2}$ and/or $g_{m4}$ and the FO can be controlled electronically by $g_{mf}$.

## 4. Simulation Results

The circuit was designed and simulated using the Cadence Virtuoso Analog Design Environment with 130 nm using UMC CMOS technology. The transistor’s aspect ratio and the values of passive devices are shown in Table 2. The voltage supply was 0.3 V (±0.15 V), the bias current of the DDCC I_B_ = 50 nA, and the nominal value of the setting current of the TA I_set_ = 0.5 µA. The nominal power consumption of the DDTA was 357.4 nW. The input and compensation capacitors were highly linear metal–isolator–metal capacitors (MIM). The linear resistor’s *R* was a high-resistance poly-resistor. Intensive simulation results of the standalone MI-DDTA including Monte Carlo analysis (MC) and process, voltage, and temperature (PVT) corners were presented in detail in [10]. The open-loop gain of the DDCC (i.e., without the unity gain feedback) was simulated as 73.9 dB and the phase margin was 56.2° for 20 pF load capacitor. The low-frequency gain for *V*_*w*_/*V*_*y*+1_ (=*V*_*w*_/*V*_*y*+2_) and *V*_*w*_/*V*_*y*-1_ (=*V*_*w*_/*V*_*y*-2_) is 14 mdB and 57.29 mdB while the −3 dB bandwidth is 22.24 kHz and 22.23 kHz, respectively. The simulated DC transfer characteristics of the DDCC show rail-to-rail operation capability. The simulated gain and phase characteristics for the TA with I_set =_ 0.5 µA and 20 pF load capacitance show that the low DC gain is 23.2 dB and the bandwidth is 19.65 kHz, while the phase error is 3.8° [10]. The DC characteristic of the output current and the transconductance of the TA versus fully differential input voltage for I_set_ = 0.125 µA, 0.25 µA, 0.5 µA confirms a rail-to-rail operation with high linearity.

**Figure 6:**
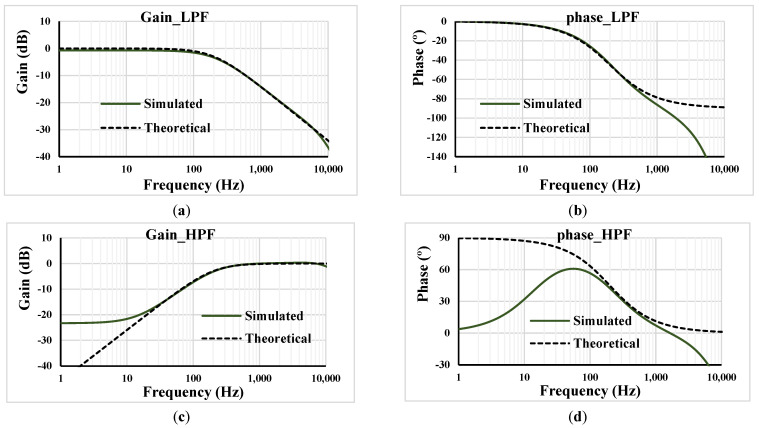

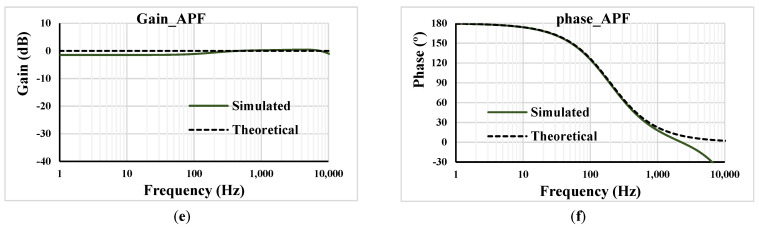
Frequency characteristics of the gain and phase: (**a**,**b**) LPF; (**c**,**d**) HPF; (**e**,**f**) APF.

Figure 6 shows the simulated and theoretical frequency characteristics of the gain and phase of the proposed voltage-mode first-order universal filter from Figure 4 with *R*_1_ = 400 kΩ, capacitor *C*_1_ = 2 nF and setting current I_set1_ = I_set2_ = 0.5 µA (*g*_*m*_ = 2.5 µS). The designed cut-off frequency of 197.4 Hz was very close to the simulated frequency of 195.6 Hz.

**Figure 7:**
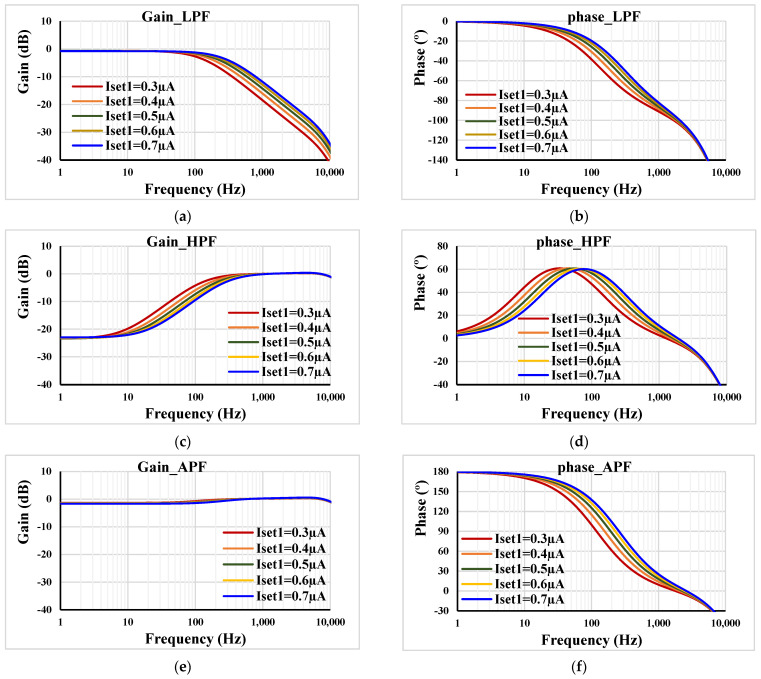
Frequency responses of the gain and phase: (**a**,**b**) LPF; (**c**,**d**) HPF; (**e**,**f**) APF with different I_set1_.

In order to confirm the electronic tuneability of the filter, the frequency characteristics of the gain and phase were repeated for *C*_1_ = 2 nF, *R*_1_ = 400 kΩ, I_set2_ = 0.5 µA, and different I_set1_ = (0.3, 0.4, 0.5, 0.6, 0.7) µA. the cut-off frequency was 120.8 Hz, 158.8 Hz, 195.6 Hz, 231.8 Hz, 260.7 Hz, respectively, as depicted in Figure 7.

**Figure 8:**
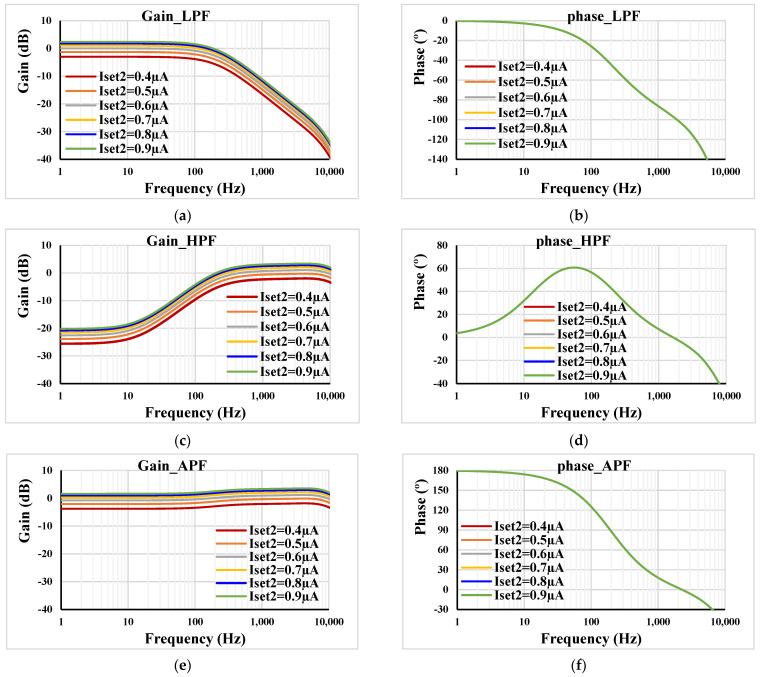
Frequency responses of the gain and phase: (**a**,**b**) LPF; (**c**,**d**) HPF; (**e**,**f**) APF with different I_set2_.

Figure 8 shows the frequency characteristics of the gain and phase for *C*_1_ = 2 nF, *R*_1_ = 400 kΩ, I_set1_ = 0.5 µA, and different I_set2_ = (0.4, 0.5, 0.6, 0.7, 0.8, 0.9) µA. The gain was in range of −3 dB to 3.3 dB, which confirms the electronic tunability of the filter gain. When the value of the resistor was changed to *R*_1_ = 800 kΩ, the gain was in range of −3 dB to 8 dB.

**Figure 9:**
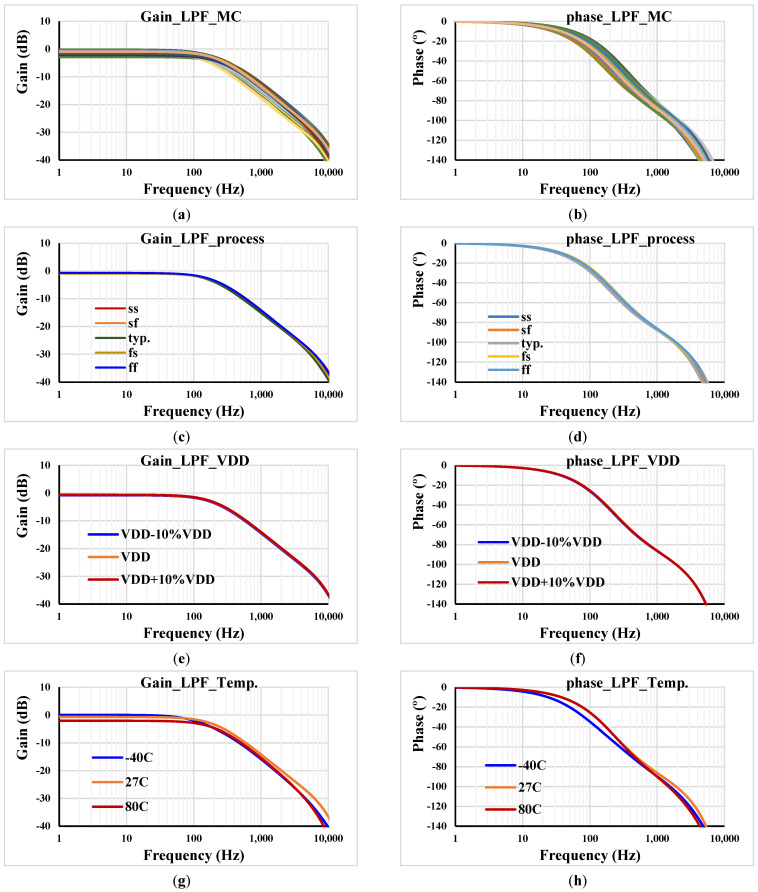
Frequency responses of the gain and phase for LPF: (**a**,**b**) MC; (**c**,**d**) process corners; (**e**,**f**) voltage corners and (**g**,**h**) temperature corners.

Figure 9 shows the frequency characteristics of the gain and phase of the LPF for *C*_1_ = 2 nF, *R*_1_ = 400 kΩ, and I_set1,2_ = 0.5 µA. The Monte Carlo analysis, including process and mismatch variation and 200 runs, is shown in (a,b). The gain of the LPF at low frequency was in the range of −2.87 dB to −0.243 dB and the cut-off frequency was in the range of 158.48 Hz to 335.26 Hz. The slow–slow (ss), slow–fast (sf), fast–slow (fs) and fast–fast (ff) processes are shown in (c,d). Finally, (e,f) shows the voltage supply corners, V_DD_ ± 10%V_DD_, and (g,h) shows the temperature corners −40 °C and 80 °C. As is evident, the curves are almost overlapped, with a slight variation in the temperature corner analysis. This is suspected to be due to the subthreshold region operation that is known to be sensitive to temperature. The deviation of the cut-off frequency can be readjusted by the setting current.

**Figure 10:**
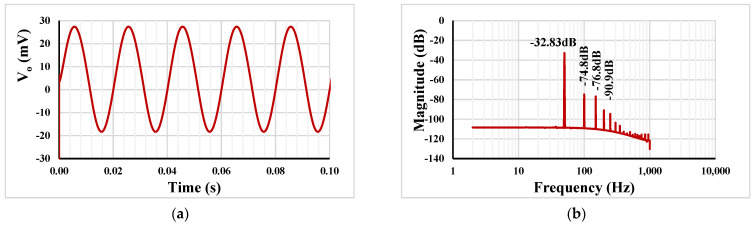
The transient response of the LPF (**a**) and the spectrum of the *V*_o_ using FFT (**b**).

Figure 10a shows the transient response of the LPF with *R*_1_ = 400 kΩ, capacitor *C*_1_ = 2 nF, the setting current I_set1_ = I_set2_ = 0.5 µA, and the applied sine wave input signal 50 mV_pp_@ 50 Hz. The spectrum of the output signal *V*_*o*_ using fast Fourier transform (FFT) is shown in (b). The total harmonic distortion was calculated as −39.97 dB (1.024%).

The comparison between the proposed versatile first-order filter and some previous works is shown in Table 3. Recently published filters, such as the CM filter in [29], the VM filters in [44,46,51,59], or the MM filter in [54], have been selected for comparison. The proposed filter offers six transfer functions similar to [29], but the proposed filter can control the voltage gain of the transfer functions. Moreover, the CM filter in [29] uses multiple-output ICCII, which has a high-power consumption. Compared with [44,46,51,59], the proposed filter exhibits rich filtering functions, and the gain of all transfer functions can be controlled. The MM filter in [54] offers nine transfer functions through CM, VM, TAM, and TIM filters, but each filter offers only three transfer functions of LP, HP, and AP filters, whereas the proposed VM filter offers six transfer functions of LP, HP, and AP filters. The filters using a single active device in [44,46,54] apply the input voltage signal via a capacitor or resistor, which is not ideal for voltage-mode circuits and integrated circuits. In comparison, the proposed filter has high-input and low-output impedance. Finally, compared with all filters in [29,44,46,51,54,59], the proposed filter unequivocally offers the lowest power supply and power consumption, which is served by the low-voltage low-power MI-DDTA. The comparison table also includes a figure of merit (FoM) = P_diss_/(N) *f*_o_, where P_diss_ is the power dissipation, N is the filter order (number of poles), and *f*_o_ is the pole frequency. However, here it is important to note that this FoM is specific to power dissipation, filter order, and pole frequency and does not cover all aspects of filter performance. The proposed filter offers many advantages over other designs due to the multiple-input MOS transistor, which increases the capability of the arithmetic operations of the proposed MI-DDTA without increasing the power consumption such as number of filter functions, high input impedance, electronic parameter control, gain control, and use of grounded passive components, which is the main attractive performance of the proposed filter which cannot be achieved by using two conventional DDTAs.

**Figure 11:**
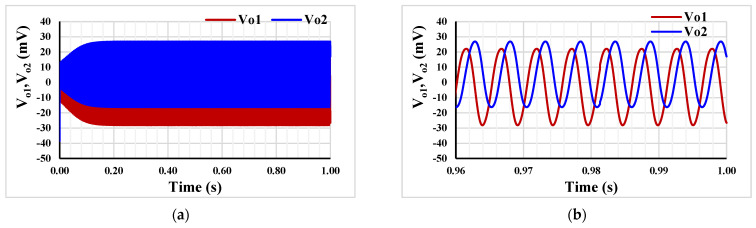
The running oscillation (**a**) and the steady state (**b**).

**Figure 12:**
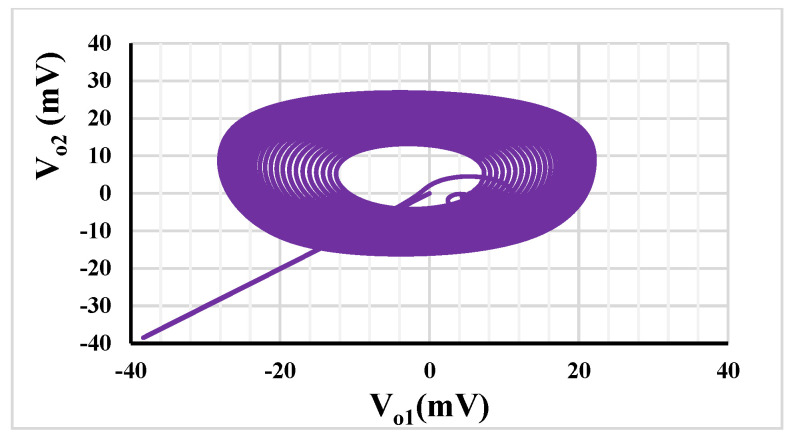
The quadrature relationship between *V*_*o*1_ and *V*_*o*2_.

For the oscillator in Figure 5, *R*_1_ = 400 kΩ, capacitors *C*_1_ = *C*_2_ = 2 nF and setting current I_set1_ = 0.5 µA were selected. To start the oscillation, *R*_2_ = 500 kΩ and I_set2_ = 0.55 µA. Figure 11a,b show the running oscillation and steady state, respectively. The THD was around 3%. The outputs *V*_*o*1_ and *V*_*o*2_ are in quadrature with a frequency of 192 Hz. Figure 12 shows the relation between *V*_*o*1_ and *V*_*o*2_ that can confirm the quadrature relationship of the output signals.

## 5. Conclusions

This work presents the realization of a versatile first-order analog filter that consists of two MI-DDTAs, one grounded capacitor, and one resistor. The proposed filter possesses six transfer functions of LP, HP, and AP filters in a single topology, high-input and low-output impedance, and electronic control of the gain and the pole frequency of transfer functions. Thanks to the bulk-driven multiple-input MOS transistor technique operating in the subthreshold region, the DDTA works with a 0.3 V of power supply and 357.4 nW of power consumption. In order to confirm the advantages of the new circuit, the proposed first-order filter has been used to realize a sinusoidal oscillator with a frequency of 192 Hz and 3% THD. The filter and oscillator are suitable for low frequency applications such as biomedical applications. The simulation results confirm the performance of the filter.

## Figures and Tables

**Table 1:** Obtaining variant filtering functions of the first-order analog filter.

Filtering Function		Input	Transfer Function	
LP	Non-inverting	$V_{in1}=V_{in}$	$\frac{V_{o1}}{V_{in}}=\frac{g_{m1}}{sC_{1}+g_{m1}}$	$\frac{V_{o2}}{V_{in}}=g_{m2}R_{1}\left(\frac{g_{m1}}{sC_{1}+g_{m1}}\right)$
	Inverting	$V_{in2}=V_{in}$	$\frac{V_{o1}}{V_{in}}=-\frac{g_{m1}}{sC_{1}+g_{m1}}$	$\frac{V_{o2}}{V_{in}}=-g_{m2}R_{1}\left(\frac{g_{m1}}{sC_{1}+g_{m1}}\right)$
HP	Non-inverting	$V_{in3}=V_{in}$	$\frac{V_{o1}}{V_{in}}=\frac{sC_{1}}{sC_{1}+g_{m1}}$	$\frac{V_{o2}}{V_{in}}=g_{m2}R_{1}\left(\frac{sC_{1}}{sC_{1}+g_{m1}}\right)$
	Inverting	$V_{in4}=V_{in}$	$\frac{V_{o1}}{V_{in}}=-\frac{sC_{1}}{sC_{1}+g_{m1}}$	$\frac{V_{o2}}{V_{in}}=-g_{m2}R_{1}\left(\frac{sC_{1}}{sC_{1}+g_{m1}}\right)$
AP	Non-inverting (Phase leg)	$V_{in2}=V_{in3}=V_{in}$	$\frac{V_{o1}}{V_{in}}=\frac{sC_{1}-g_{m1}}{sC_{1}+g_{m1}}$	$\frac{V_{o2}}{V_{in}}=g_{m2}R_{1}\left(\frac{sC_{1}-g_{m1}}{sC_{1}+g_{m1}}\right)$
	Inverting (Phase lead)	$V_{in1}=V_{in4}=V_{in}$	$\frac{V_{o1}}{V_{in}}=-\frac{sC_{1}-g_{m1}}{sC_{1}+g_{m1}}$	$\frac{V_{o2}}{V_{in}}=-g_{m2}R_{1}\left(\frac{sC_{1}-g_{m1}}{sC_{1}+g_{m1}}\right)$

**Table 2:** Transistor aspect ratios of the MI-DDTA.

Device	W/L (µm/µm)
M_1A_, M_2A_, M_1B_, M_2B_	20/3
M_7_, M_8_	15/3
M_3_–M_6_, M_B_	10/3
M_9_	6 × 10/3
M_10_	6 × 20/3
M_R_	5/3
MIM capacitor: *C*_*B*_ = 0.2 pF, *C*_*c*_ = 4 pF	
Poly-resistor *R* = 90 kΩ	

**Table 3:** Comparison of the proposed design with previous first-order filters.

Features	Proposed	[29] 2017	[44] 2021	[46] 2022	[51] 2022	[54] 2023	[59] 2023
Active and passive elements	2 DDTA, 1 C, 1 R	2 ICCII, 1 C, 1 MOS Figure 4	1 LT1228, 1 C, 2 R	1 VD-DIBA, 1 C, 2 R	2 CFOA, 1 C, 4 R Figure 1d	1 VGA, 1 C, 1 R	2 OTA, 1 C
Realization	CMOS structure (0.13 μm)	CMOS structure (0.13 μm)	Commercial IC (LT1228)	Commercial IC (LT1228, AD830)	Commercial IC (AD844)	CMOS structure (0.18 μm)	CMOS structure (0.18 μm)
Mode operation	VM	CM	VM	VM	VM	MM	VM
Type of filter	MISO	SIMO	MISO	MISO	MISO	MIMO	MISO
Number of filtering functions	6 (LP+, LP−, HP+, HP−, AP+, AP−)	6 (LP+, LP−, HP+, HP−, AP+, AP−)	4 (LP+, HP+, AP+, AP−)	4 (LP−, HP−, AP+, AP−)	3 (LP−, HP+, AP+)	3 (LP+, HP+, AP+)	6 (LP+, LP−, HP+, HP−, AP+, AP−)
High-input impedance	Yes	-	No	No	Yes	No	Yes
Electronic control of parameter $\omega_{o}$	Yes	Yes	Yes	Yes	No	Yes	Yes
Control of gain	Yes	No	Yes (LP, HP)	Yes (LP, HP)	Yes	No	No
Using grounded capacitor/resistor	Yes	No	No	No	No	No	Yes
Pole frequency (kHz)	0.195	2600	90	159.15	159	1590	0.220
Total harmonic distortion (%)	1.024@50 mV_pp_	<1.5@90 μA_pp_	1@200 mV_pp_	<1@150 mV_pp_	-	-	0.36@40 mV_pp_
Supply voltage (V)	0.3	±0.75	±5	±5	±12	±0.9	0.5
Power dissipation (µW)	0.7148	4080	57,600	-	-	-	59.5 × 10^−3^
FoM (µW/kHz)	3.66	1.56	640	-	-	-	0.27
Verification of result	Sim.	Sim.	Exp.	Exp.	Exp.	Sim/Exp	Post-layout Sim.

**Note**: VD-DIBA = voltage differencing differential input buffered amplifier, ICCII = inverting second-generation current conveyors, VGA = voltage differencing gain amplifier, MISO = multiple-input single-output, SIMO = single-input multiple-output, MIMO = multiple-input multiple-output, MM = Mixed-mode, LP+ = non-inverting low-pass filter, LP− = inverting low-pass filter, HP+ = non-inverting high-pass filter, HP− = inverting high-pass filter, AP+ = non-inverting all-pass filter, AP− = inverting all-pass filter.
